# New possibilities for neuroprotection in neonatal hypoxic-ischemic encephalopathy

**DOI:** 10.1007/s00431-021-04320-8

**Published:** 2021-11-24

**Authors:** Suresh Victor, Eridan Rocha-Ferreira, Ahad Rahim, Henrik Hagberg, David Edwards

**Affiliations:** 1 Centre for the Developing Brain, Department of Perinatal Imaging and Health, School of Biomedical Engineering and Imaging Sciences, King’s College London, 1st Floor, South Wing, St Thomas’ Hospital, Westmister Bridge Road, London, UK; 2 Centre for Perinatal Medicine and Health, Institute of Clinical Sciences, Sahlgrenska Academy, University of Gothenburg, Gothenburg, Sweden; 3 UCL School of Pharmacy, University College London, London, UK

**Keywords:** Infant, Newborn, Brain, Encephalopathy, Neuroprotection, Hypothermia

## Abstract

Around 0.75 million babies worldwide suffer from moderate or severe hypoxic-ischemic encephalopathy (HIE) each year resulting in around 400,000 babies with neurodevelopmental impairment. In 2010, neonatal HIE was associated with 2.4% of the total Global Burden of Disease. Therapeutic hypothermia (TH), a treatment that is now standard of care in high-income countries, provides proof of concept that strategies that aim to improve neurodevelopment are not only possible but can also be implemented to clinical practice. While TH is beneficial, neonates with moderate or severe HIE treated with TH still experience devastating complications: 48% (range: 44–53) combined death or moderate/severe disability. There is a concern that TH may not be effective in low- and middle-income countries. Therapies that further improve outcomes are desperately needed, and in high-income countries, they must be tested in conjunction with TH. We have in this review focussed on pharmacological treatment options (e.g. erythropoietin, allopurinol, melatonin, cannabidiol, exendin-4/exenatide). Erythropoietin and allopurinol show promise and are progressing towards the clinic with ongoing definitive phase 3 randomised placebo-controlled trials. However, there remain global challenges for the next decade.

*Conclusion*: There is a need for more optimal animal models, greater industry support/sponsorship, increased use of juvenile toxicology, dose-ranging studies with pharmacokinetic-pharmacodynamic modelling, and well-designed clinical trials to avoid exposure to harmful medications or abandoning putative treatments.

**Table:** 

**What is Known:** *• Therapeutic hypothermia is beneficial in neonatal hypoxic-ischemic encephalopathy.* *• Neonates with moderate or severe hypoxic-ischemic encephalopathy treated with therapeutic hypothermia still experience severe sequelae.*	
**What is New:** *• Erythropoietin, allopurinol, melatonin, cannabidiol, and exendin-4/exenatide show promise in conjunction with therapeutic hypothermia.* *• There is a need for more optimal animal models, greater industry support/sponsorship, increased use of juvenile toxicology, dose-ranging studies with pharmacokinetic-pharmacodynamic modelling, and well-designed clinical trials.*

**What is Known:**

*• Therapeutic hypothermia is beneficial in neonatal hypoxic-ischemic encephalopathy.*

*• Neonates with moderate or severe hypoxic-ischemic encephalopathy treated with therapeutic hypothermia still experience severe sequelae.*

**What is New:**

*• Erythropoietin, allopurinol, melatonin, cannabidiol, and exendin-4/exenatide show promise in conjunction with therapeutic hypothermia.*

*• There is a need for more optimal animal models, greater industry support/sponsorship, increased use of juvenile toxicology, dose-ranging studies with pharmacokinetic-pharmacodynamic modelling, and well-designed clinical trials.*

## Introduction

Hypoxic-ischemic encephalopathy (HIE) is the neurological syndrome that occurs in the newborn infant subject to different degrees of a hypoxic-ischemia event. It is associated with loss of consciousness; decrease of spontaneous movements, tone, and reflexes; and appearance of convulsions in the more severe cases. HIE is a global problem with an estimated incidence of 1.5–2.0 per 1000 live births [1, 2]. Around 0.75 million babies worldwide suffer from moderate or severe HIE each year resulting in around 400,000 babies with neurodevelopmental impairment. HIE was associated with 2.4% of the total Global Burden of Disease (2010) [3]

Therapeutic hypothermia (TH), a treatment strategy that is now standard of care in high-income countries, provides proof of concept that treatment strategies that aim to improve neurodevelopment are not only possible but can be implemented to clinical practice [4]. In a meta-analysis of 7 trials, representing 1214 newborn infants, TH reduced the risk of the composite outcome of death or major neurodevelopmental disability at age 18 months (risk ratio (RR), 0.76; 95% confidence interval (CI), 0.69–0.84; and numbers needed to treat (NNT), 7; 95% CI, 5–10) [5]. While TH is beneficial, neonates with moderate or severe HIE treated with TH still experience devastating complications: mortality 28% (24–38); cognitive impairment 24% (21–25); cerebral palsy 22% (13–28); epilepsy 19% (15–24); cortical visual impairment 6% (1–10), with combined death or moderate/severe disability 48% (44–53) [5]. Adjunctive therapies to further improve outcomes are desperately needed. In high-income countries, experimental therapies must be tested in conjunction with the standard of care: TH.

TH requires a high level of intensive care support, and this cannot always be provided in low- and middle-income countries. Furthermore, TH has been shown to be ineffective and possibly even harmful in the presence of infection/inflammation [6, 7].

There remains a need to develop newer neuroprotective treatment strategies. This review will compare emerging treatments in terms of efficacy in preclinical experiments, safety profile in humans, and toxicology experiments and anticipated effect size.

## Erythropoietin

Erythropoietin is a cytokine with multiple roles in addition to haemopoietic growth factor. Erythropoietin receptors are in neurons, glia, and endothelial cells [8, 9]; they participate in proliferation and differentiation of these cells both during normal brain development and following hypoxia [10–14]. Hypoxia and pro-inflammatory cytokines activate hypoxia-inducible factor to induce expression of erythropoietin and receptors [15]. Erythropoietin provides neuroprotection by promoting anti-apoptotic, anti-oxidative, and anti-inflammatory responses [16, 17]. Additionally, erythropoietin increases neuronal and glial migration around the injured area via the secretion of matrix metalloproteinases [18].

### Preclinical evidence

Much of the evidence supporting the use of erythropoietin as a neuroprotective agent in neonatal HIE was determined in rodent models with no added hypothermia [12, 17, 19–23]. In a nonhuman primate model of HIE (15–18-min occlusion of umbilical artery in *Macaca nemestrina*), intravenous erythropoietin (3500 U/kg × 1 dose followed by 3 doses of 2500 U/kg, or 1000 U/kg/day × 4 doses) was administered on days 1, 2, 3, and 7 and combined with 72 h hypothermia [24]. Erythropoietin improved motor and cognitive responses, cerebellar growth, and diffusion imaging measures and produced a death/disability (cerebral palsy at 9 months of age) relative risk reduction of 0.911 (95% CI − 0.429 to 0.994), an absolute risk reduction of 0.395 (95% CI 0.072–0.635), and a NNT of 2 (95% CI 2–14) when compared with no treatment [24]. Neuropathology was significantly decreased at 9 months of age [25].

In a piglet model of HIE (bilateral carotid artery ligation with exposure to 8–10% oxygen), erythropoietin (3000 U/kg) was administered as an intravenous bolus at 1 h, 24 h, and 48 h in conjunction with 12 h hypothermia [26]. The area under curve (AUC) target therapeutic concentrations of 117,677–140,331 U * h/L was achieved. Erythropoietin and hypothermia double therapy had no effect on brain lactate/N-acetyl aspartate (Lac/NAA) ratio on Magnetic Resonance Spectroscopy (MRS) or Terminal deoxynucleotidyl transferase dUTP nick end labelling-positive (TUNEL-positive) cells (a measure of apoptosis), but there was more rapid amplitude-integrated electroencephalography (aEEG) recovery from 25 to 30 h and increased oligodendrocyte survival [26].

### Pharmaceutics and licenced preparation (Table 1)

**Table 1:** Pharmaceutics and licenced dose*

Nonproprietary name	Trade name and maximum dose	Generic/biosimilar version approved	Licenced indication in children	Excipients of significance	Common side effects of active ingredient	Known side effects of excipient
Recombinant erythropoietin (Epoetin Alfa)	Epogen® (Amgen®, USA) 150 units/kg intravenous thrice weekly in children < 10 kg	Yes	Symptomatic anaemia in chronic renal failure	Benzyl alcohol	Arthralgia; embolism and thrombosis; headache; hypertension (dose-dependent); influenza-like illness; skin reactions; stroke	Benzyl alcohol administered intravenously in the range of 100 to 200 mg/kg/day has been linked to the “gasping syndrome” [116]
Melatonin	Slenyto® (Neurim Pharmaceuticals®, Israel) 10 mg/day oral in children > 2 years No intravenous preparation—poor water solubility	Not in UK and Europe Sold over the counter in the USA	Insomnia with autism spectrum disorder or Smith-Magenis syndrome	In animal experiments: ethanol (Sigma-Aldrich®, USA) and ethanol-free (Chiesi Farmaceutici®, Italy)	Arthralgia; abnormal behaviour; drowsiness; feeling abnormal; headaches; increased risk of infection; altered mood; pain; sleep disorders	Ethanol-free formulation required
Cannabidiol	Epidyolex® (GW Pharmaceuticals®, UK) 20 mg/kg/day oral in children > 2 years No intravenous preparation—poor water solubility	No	Seizures associated with Lennox-Gastaut syndrome or Dravet syndrome	In animal experiments: Ethanol (GW Research Ltd.)	Agitation; appetite abnormal; abnormal behaviour; cough; diarrhoea; drooling; drowsiness; fatigue; fever; increased risk of infection; insomnia; irritability; rash; tremor; vomiting; weight decreased	Ethanol-free formulation undergoing clinical trial
Exenatide/exendin-4	Byetta® (AstraZeneca®, UK) 10 μg twice daily subcutaneous in adults	Yes	Type 2 diabetes mellitus for glycaemic control	Metacresol	Appetite decreased; asthenia; constipation; diarrhoea; dizziness; gastrointestinal discomfort; gastrointestinal disorders; headache; nausea; skin reactions; vomiting	Metacresol at high doses may be cytotoxic and pro-inflammatory [117] Metacresol-free formulation is required
Allopurinol sodium	Aloprim® (Mylan Institutional®, USA) 200 mg/m^2^/day intravenous. Lyophilised powder reconstituted in saline	Yes	Prophylaxis for hyperuricaemia induced by chemotherapy	Sodium hydroxide	Rash	Irritation to the skin, eyes, mucous membranes (high concentrations), toxic pneumonitis; dermatotoxin; and dysphagia

^*^Source: US Food and Drug Administration (FDA) and European Medicines Agency (EMA)

Recombinant human erythropoietin was used in most animal experiments and clinical trials [27].

### Dosing and pharmacokinetics

In a dose-escalating, safety, and tolerance study, the AUC target therapeutic concentrations of 131,054 U * h/L were achieved using intravenous erythropoietin 1000 U/kg [28, 29]. The half-life at this dose was 15.7 h [29]. Clinical trials with dose < 1000 U/kg [30–33], single dosing [34], and repeat dosing with > 24-h intervals [30] may have not achieved maximum therapeutic benefit. Although efficacy has been demonstrated in animal models using subcutaneous dosing regimens, an intravenous dosing regimen where peak concentrations are rapidly achieved is more likely to achieve maximum therapeutic benefit [33–36]. In animal experiments, both immediate and delayed treatments are effective [27].

### Safety and toxicology

Common side effects in adults and children are shown in Table 1. Sufficiently, high dose range has not been studied in toxicology experiments. Repeat dose toxicity (13 weeks) was noted at lower dose than anticipated neonatal dose (Table 2).

**Table 2:** Toxicology*

Nonproprietary name	Neonatal HIE dose (based on animal studies with hypothermia)	Trade name under which toxicology performed	Toxicology: species, dose range, and route	Juvenile toxicology	Significant toxicology results
Recombinant erythropoietin/epoetin alfa	1000 U/kg intravenous on PNDs 1, 2, 3, and 7	Epogen® (Amgen®, USA)/Epoetin Hospira® (Hospira Inc.®, USA)	1500 U/kg (human equivalent dose of 833 U/kg) intravenous three times weekly for 13 weeks in dogs with recovery for 4 weeks. No single-dose toxicity studies reported	No studies	Hypoactivity, loss of limb function (1 male), discoloration of the faeces, reduced faecal output, dehydration, red gums and/or discoloration (yellow) of the teeth; 3 of 4 recovery remained thin throughout 4-week recovery period
Melatonin	10 mg/kg intravenous on PNDs 1 and 2	Circadin®(Neurim Pharmaceuticals®, Israel)/Slenyto®(Neurim Pharmaceuticals®, Israel)	Only single-dose toxicity studies using intravenous route reported. Dose range not reported	No studies	The intravenous lethal dose (LD_50_) is 180 to 472 mg/kg in mice and 356 mg/kg in rats (human equivalent dose: 15 to 58 mg/kg). The higher doses led to sedation, lethargy, impairment of righting, placing and flexor reflexes, marked reduction in body temperature, and respiratory distress preceding death
Cannabidiol	0.3 mg/kg intravenous on PND 1. Further dose-ranging studies are required	Epidyolex® (GW Pharmaceuticals®, UK)	See juvenile toxicology	Subcutaneous doses of 0 or 15 mg/kg on postnatal days 4–6 followed by oral administration of 0, 100, 150, or 250 mg/kg on PNDs 7–77 in rats	Increased body weight, delayed male sexual maturation, neurobehavioural effects, increased bone mineral density, and liver hepatocyte vacuolation. The lowest dose causing developmental toxicity was 15 mg/kg subcutaneous (human equivalent dose of 1 mg/kg) in juvenile rats
Exenatide/exendin-4	Dose-ranging studies are required Anticipated 90 μg/kg 12 hourly intravenous (equivalent)	Byetta® (AstraZeneca®, UK)	Subcutaneous doses of 100, 300, 1000, 3000, and 5000 μg/kg in monkeys	No studies	No mortality or signs of serious toxicity at any dose. Doses ≥ 5000 μg/kg (human equivalent dose of 1600 μg/kg) caused decreased food consumption
Allopurinol sodium	Dose-ranging studies are required	Aloprim® (Mylan Institutional®, USA)	No dose-ranging toxicology reported	No studies	In mice, the minimal lethal dose is 45 mg/kg intravenous (human equivalent dose of 3.65 mg/kg). Hypoactivity was observed with these doses. In rats, the minimum lethal dose is 100 mg/kg intravenous (human equivalent dose of 16 mg/kg)

^*^Source: EMA and FDA; human equivalent dose was calculated according to FDA guidance [118]

### Clinical trials

Safety and tolerance was established in early phase clinical trials [37, 38]. In a phase 2 randomised placebo-controlled trial on 50 infants [39], intravenous erythropoietin (1000 U/kg) on postnatal days 1 (< 24 h after birth), 2, 3, and 5 showed a lower global brain injury score in erythropoietin-treated infants (median, 2 vs. 11, *P* = 0.01) on brain magnetic resonance imaging (MRI) done at mean 5.1 days (SD, 2.3). Moderate/severe brain injury (4% vs. 44%, *P* = 0.002), subcortical (30% vs. 68%, *P* = 0.02), and cerebellar injury (0% vs. 20%, *P* = 0.05) were less frequent in the erythropoietin group than in the placebo group.

Two ongoing phase 3 randomised placebo-controlled trials (NCT01732146; NCT02811263) aim to determine the effect of intravenous erythropoietin on death and disability at 24 months in infants with moderate or severe HIE [40]. The HEAL trial (NCT02811263; study completion September 2022) is evaluating the efficacy of intravenous erythropoietin (1000 U/kg) on postnatal days 1 (< 24 h after birth), 2, 3, 4, and 7 on 500 participants. The sample size is based on an anticipated 15–20% reduction in the combined outcome of death and disability [40]. The NUREPO trial (NCT01732146) is evaluating the efficacy of intravenous erythropoietin (1000–1500 U/kg) on postnatal days 1 (< 12 h after birth), 2, and 3 on 120 participants (completed 2017; not reported).

## Melatonin

Melatonin (5-methoxy-N-acetyltryptamine) is produced by the pineal gland according to a circadian cycle [41]. It acts through three receptors (MT1, MT2, and MT3), highly expressed in the foetal brain and leptomeninges, where it plays a role in brain growth and development [42–44].

Melatonin acts as a direct and indirect antioxidant, being a potent scavenger of superoxide anion and stimulator of the synthesis of antioxidant enzymes [45]. Melatonin achieves neuroprotective effect via antioxidant, anti-apoptotic, and anti-inflammatory processes and by promoting neuronal and glial development [46, 47].

### Preclinical evidence

The neuroprotective effect of intravenous melatonin (15–30 mg/kg) on postnatal days 1 (1–6 h after birth) and 2 has been assessed in a piglet model of HIE (bilateral carotid artery ligation with exposure to hypoxia) in conjunction with 12–24 h hypothermia [26, 48–50]. The target concentrations (maximum concentrations (*C*_max_): 16.8 ± 8.3 mg/L and AUC: 555 ± 266 mg ∗ h/L) were achieved at 15-mg/kg dose. However, reduction in brain Lac/NAA ratios was achieved at 18-mg/kg and 30-mg/kg doses. Reduction in TUNEL-positive cell death was achieved in the hippocampus, caudate nucleus, internal capsule, and putamen at 30 mg/kg dose (with ethanol) and only in sensory cortex at the 20 mg/kg (without ethanol).

In a lamb model of HIE (umbilical cord occlusion for 9–10 min), intravenous melatonin (with ethanol) was administered at 15 mg/kg/day in 12 divided doses every 2 h; a steady plasma concentration of melatonin and cortical concentration of 0.46 ± 0.16 ng/mL were achieved [51]. Melatonin improved all neurodevelopmental assessments and reduced seizure burden. Significant reduction in brain Lac/NAA ratio and apoptosis was also achieved suggesting that alternative dosing approaches such as continuous infusion may also be effective [51].

### Pharmaceutics and licenced preparation (Table 1)

Melatonin is photosensitive and degrades rapidly within hours of UV-A and UV-B exposure, necessitating specific storage and administration requirements [49].

### Dosing and pharmacokinetics

Pharmacokinetic modelling in piglets indicates that a dose of 20–30 mg/kg intravenous for 2 h repeated 24 h later is required to maintain a therapeutic concentration of 15–30 mg/L [52]. Detailed pharmacokinetic studies are required in neonates with moderate and severe HIE before a dosing regimen is determined. Target therapeutic concentrations need to be achieved within 3 h after birth.

### Safety and toxicology

Oral melatonin has been safely administered to children [53], pregnant women [54–56], and preterm newborn infants [57] at much lower doses with no safety concerns (Table 1). The intravenous lethal dose (LD_50_) in rodents without anaesthesia is not much higher than the anticipated treatment dose (Table 2). Intensive care may be required for treatment.

### Clinical trials

Small trials have been conducted with enteral melatonin with [58] or without hypothermia [59–61]. A clinical trial using low-dose enteral melatonin (0.5–5 mg/kg) is currently recruiting (NCT02621944; study completion: March 2022). In a randomised placebo-controlled trial on infants with moderate-severe HIE (*n* = 25), intravenous melatonin (5 mg/kg as 2 h infusion) on postnatal days 1 (< 6 h after birth), 2, and 3 only improved the cognitive composite score (101 ± 22 vs. 86 ± 17; *p* < 0.05) on Bayley Scales of Infant Development III at 18 months of age [62]. There were no differences between the groups according to the Gross Motor Function Classification System.

A well-designed early-phase escalating dose clinical trial is required to determine the pharmacokinetics, safety, and tolerance of intravenous melatonin.

## Cannabidiol

Cannabidiol is one of the naturally occurring cannabinoids found in cannabis plants. It is a 21-carbon terpenophenolic compound which is formed following decarboxylation from a cannabidiolic acid precursor, although it can also be produced synthetically. It has a complex pharmacological profile, acting not only on endocannabinoid receptors, CB1 and CB2, but also on G protein–coupled receptors, ion channel, and nuclear receptors [63, 64]. While some of the neuroprotective effects of cannabidiol are mediated through CB1 and CB2, it is also partly due to activation of 5-hydroxytryptamine-1A, adenosine, and peroxisome proliferator-activated receptor-gamma (PPARγ) receptors [65–68]. It reduces apoptosis and mitochondrial dysfunction and acts as an antioxidant by reducing the activity of the antioxidant system and increasing the activity of mitochondrial complexes [65, 69, 70]. It reduces inflammation by decreasing pro-inflammatory cytokine production and increasing anti-inflammatory cytokine production and stimulation of PPARγ [65, 71].

### Preclinical evidence

Cannabidiol was administered to piglets as an intravenous bolus (1 mg/kg) on postnatal days 1 (30 min after hypoxic-ischemic insult), 2, and 3 in conjunction with 48 h hypothermia [72]. The hypoxic-ischemic insult (bilateral carotid artery ligation with exposure to 10% oxygen) induced increases in brain Lac/NAA ratio, and TUNEL-positive cells in the cerebral cortex were reversed by combined hypothermia and cannabidiol but not by either alone. No treatment modified the effects of hypoxic ischemia on oxidative stress, astroglial activation, background electroencephalography, or seizures [72].

The neuroprotective effect of combined intravenous cannabidiol (1 mg/kg, 30 min after hypoxic-ischemic insult) and hypothermia (6 h) was studied using a piglet HIE model (bilateral carotid artery occlusion with exposure to < 10% oxygen). Individually, hypothermia and the cannabidiol treatments reduced the number of necrotic neurons and prevented an increase in Lac/NAA ratio [73]. The combined effect of hypothermia and cannabidiol on excitotoxicity, on inflammation and oxidative stress, and on cell damage was greater than either hypothermia or cannabidiol alone [73]. In contrast, no neuroprotective effect was demonstrated when intravenous cannabidiol (1 mg/kg) and hypothermia (9 h) were administered to a global hypoxia–ischemia piglet model [74].

Low-dose cannabidiol alone (0.01 μg/kg intravenous 60 min after umbilical artery occlusion) significantly reduced TUNEL-positive cells in all brain regions (cortex, hippocampus, basal nuclei, cerebellum, brainstem) in foetal lambs at 3 h [75]. Subcutaneous cannabidiol (1 mg/kg) showed a therapeutic window of 18 h after hypoxia–ischemia in a 9–10-day-old mice using a Rice-Vanucci model (unilateral carotid artery ligation with exposure to 10% oxygen) [76].

### Pharmaceutics and licenced preparation (Table 1)

Cannabidiol is stable in room temperature and not photosensitive but has poor water solubility.

### Dosing and pharmacokinetics

Intravenous cannabidiol is rapidly distributed, followed by prolonged elimination (terminal half-life: 24 h) [77, 78]. Plasma cannabidiol concentration peaked at the end of the infusion 15 min after the end of intravenous bolus administration and rapidly decreased to low concentrations after 1 h in piglets [72]. No cumulative effect was observed after repeated doses. Hypothermia led to a significant increase in cannabidiol plasma concentration [72]. In healthy adults, mean plasma cannabidiol concentrations were reported at 686 ng/mL (3 min postadministration), which dropped to 48 ng/mL at 1 h following intravenous administration of 20 mg of deuterium-labelled cannabidiol [79].

### Safety and toxicology

Cannabidiol has very low toxicity (Table 2). Liver safety concerns were raised in randomised controlled trials of cannabidiol in patients with Lennox-Gastaut syndrome or Dravet syndrome [80, 81]. 17.2% of patients receiving up to 20 mg/kg oral cannabidiol, all taking valproic acid, had liver transaminase elevations ≥ 3 times the upper limit of normal [81]. Since the oral bioavailability of cannabidiol is only 13–19% [77], neonates with HIE receiving intravenous cannabidiol may be at risk at much lower doses.

### Clinical trials

A phase 1, escalating single dose (0.1–3 mg/kg), randomised placebo-controlled trial using a new ethanol-free intravenous formulation of cannabidiol on infants with moderate-severe HIE is currently recruiting to assess safety, tolerance, and pharmacokinetics (EudraCT Number: 2016–000,936-17; Sponsor: GW Pharmaceuticals).

## Exenatide/exendin-4

Exendin-4 is a 39 amino acid agonist of the glucagon-like peptide-1 (GLP-1) receptor. Exendin-4 is present in the saliva of the Gila monster, *Heloderma suspectum*. GLP-1, a gastrointestinal hormone secreted by the L cells of the intestine, regulates blood glucose primarily via stimulation of glucose-dependent insulin release [82]. GLP-1 agonists have neuroprotective properties when assessed in preclinical models of Alzheimer’s disease [83], Parkinson’s disease [84], traumatic brain injury [85, 86], and stroke [87, 88]. In oxygen–glucose deprivation models, GLP-1 and agonists increase neuronal survival by reducing reactive oxygen species and apoptotic and necrotic mechanisms partly through the PI3K/protein kinase B (Akt) pathway [89–91]. Exenatide readily penetrates the blood–brain barrier where it acts on GLP-1 receptors known to be present in the newborn brain [92].

### Preclinical evidence

Using a Rice-Vanucci mouse model of HIE, the potential neuroprotective effect of exendin-4 in both postnatal day 7 and 10 mice [92]. An optimal exendin-4 treatment dosing regimen was found, where four high doses (500 μg/kg intraperitoneal with TH) starting at 0 or 2 h, then at 12 h, 24 h, and 36 h after postnatal day 7 hypoxic-ischaemic insult augmented TH resulting in 80% improvement in infarct volume and cell death. Treatment with liraglutide, a long acting GLP-1 agonist, also exerted neuroprotection in a Rice-Vanucci rat model of HIE [89].

### Pharmaceutics and licenced preparation (Table 1)

Exenatide is readily soluble in water and photosensitive, necessitating special storage and administration requirements.

### Dosing and pharmacokinetics

The pharmacokinetic profile of exenatide (60–600 μg/kg) following intraperitoneal injection is like a subcutaneous injection and differs from an intravenous dose in rats. Subcutaneous bioavailability at high doses is good, but the absorption rate and clearance of exenatide is nonlinear meaning attaining rapid therapeutic concentrations may not be feasible by this route [93]. Effective therapeutic concentrations can be achieved rapidly and maintained more precisely using an intravenous bolus followed by 48 h infusion [93]. Exenatide is metabolised throughout the body, resistant to proteolytic cleavage by dipeptidyl peptidase IV, eliminated through the kidney, and unaffected by liver impairment [94].

### Safety and toxicology

Exenatide has an excellent safety profile in adults. It increases, on a glucose-dependent basis, the secretion of insulin and does not impair hormone responses to hypoglycaemia [95]. No toxicity was noted in human overdose case reports [96–98], but weight loss is a concern on repeated dosing (Table 2).

### Clinical trials

No studies have been performed in neonates. A phase 3 trial on Parkinson’s disease is currently recruiting (NCT04232969).

## Allopurinol

Allopurinol is a xanthine-oxidase inhibitor that inhibits the production of uric acid. The mechanism of neuroprotection in neonatal HIE is unclear, possibly by inhibiting the formation of the free radical superoxide production [99].

### Preclinical evidence

Several studies in rodents and piglets have shown neuroprotective effects but none have been performed in conjunction with hypothermia [100–106].

### Pharmaceutics and licenced preparation (Table 1)

Allokid® (unlicensed, ACE Pharmaceuticals®, Netherlands), specially formulated for neonates, is currently undergoing clinical trial (NCT03162653) [107]. An alternative licenced formulation, Aloprim® (Mylan Institutional®, USA), is discussed in Tables 1 and 2.

### Dosing and pharmacokinetics

Target therapeutic concentrations have not been defined in preclinical studies. Allopurinol 20 mg/kg intravenous within 2 h after birth, second dose 12 h later, then every 12 h for 3 days improved neurodevelopment at 1 year [108] and forms the basis for ongoing phase 3 trial [107]. Pharmacokinetics of 10–20 mg/kg of intravenous allopurinol was studied in neonates with HIE (*n* = 46 from 3 studies) [109]. Xanthine-oxidase inhibition was achieved, and no dose adjustment for TH was proposed [109].

### Safety and toxicology

Oral allopurinol has a good safety profile. Intravenous allopurinol has been used in association with cancer chemotherapy. An independent safety profile is difficult to establish. The intravenous lethal dose (LD_50_) in rodents without anaesthesia is not much higher than the anticipated treatment dose (Table 2).

### Clinical trials

A meta-analysis (*n* = 114 participants) did not reveal a statistically significant difference in the risk of death (typical risk ratio 0.88; 95% confidence interval (95% CI) 0.56 to 1.38; risk difference − 0.04; 95% CI − 0.18 to 0.10) or a composite of death or severe neurodevelopmental disability (typical risk ratio 0.78; 95% CI 0.56 to 1.08; risk difference − 0.14; 95% CI − 0.31 to 0.04) [110]. A phase III randomised placebo-controlled trial to evaluate the effect of postnatal allopurinol administered in addition to standard of care (including therapeutic hypothermia if indicated) on the incidence of death and disability at 24 months of age in neonatal HIE is ongoing (NCT03162653) [107].

**Table 3:**
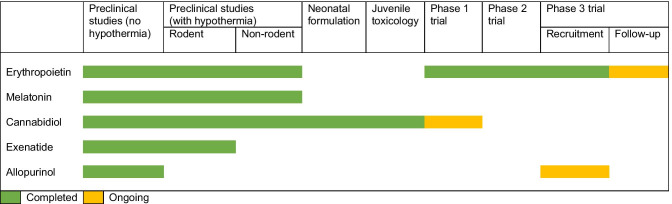
Milestones achieved in translation at the anticipated dose as described in Table 2

## Discussion

Several promising adjuvant treatment strategies for neonatal HIE are progressing towards the clinic (Table 3). We have in this review focussed on pharmacological treatment options. Stem cell therapy is outside the scope of this review. Serrenho I et al. (2021) provides a systematic review of preclinical studies done on stem cell therapy for neonatal HIE [111]. Eighty percent of these studies reported a significant improvement of cognitive and/or sensorimotor function, as well as decreased brain damage. There are no completed clinical trials on stem cell therapy, and several are ongoing [112]. Xenon [113] and magnesium sulphate [114] have been previously evaluated and did not demonstrate much benefit.

Pharmacological treatment strategies which have shown potential benefit in animal and human studies of HIE demonstrate that (1) there is a narrow time window within the first few hours after birth during which therapy should be started for best outcomes, (2) high doses are often needed and the anticipated side effects based on adult tolerability have not been noticed in newborn babies, and (3) TH changes the pharmacokinetics of drugs requiring special consideration. Key limiting factors are needed for industry support for developing new formulations suitable for use in neonates and inadequate dose range finding and pharmacokinetic studies in both animals and neonates. Allopurinol sodium and cannabidiol have received orphan designation for the treatment of neonatal HIE and only one of the ongoing clinical trials has an industry sponsor (cannabidiol; GW Pharmaceuticals).

Many treatments have progressed to human trials without juvenile toxicology. Since the proposed dose are often much higher than current licenced dose and animal models of neonatal HIE do not include a recovery period and multi-organ histopathology, the risk for serious unexpected adverse reactions remains. Juvenile toxicity studies with cannabidiol showed that lowest dose of developmental toxicity was 15 mg/kg. In contrast, the no adverse observed adverse event level (NOAEL) in adult rodents was 400–500 mg/kg emphasising the need for these studies.

Several pharmacological treatments have demonstrated efficacy in animal models. We have in this review focussed on nonrodent models of HIE. The use of unilateral/bilateral occlusion of carotid artery are well-accepted in models of neonatal HIE. However, focal ischemia models are unsuitable for assessment of pharmacokinetics. Kyng et al. (2015) describe a global hypoxia–ischemia model in piglets which may allow a more reliable preclinical assessment of target therapeutic concentration [115].

Double therapy in animal models is often tested in combination with < 48 h hypothermia. While the feasibility of maintaining ≥ 48 h hypothermia in animal experiments is low, anticipated effect sizes are unreliable with shorter duration hypothermia. Competitive advantage of different treatments cannot be determined as experiments with varying duration of hypothermia are not comparable.

Only erythropoietin has been studied in primates with follow-up included allowing for anticipated effect sizes for death or cerebral palsy as a combined outcome measure to be determined. Piglet models with no follow-up rely on surrogate biomarkers like TUNEL-positive count and Lac/NAA ratio. Adverse neurodevelopmental outcomes were correctly identified in 95.65% of cases (*n* = 62) by Lac/NAA measured using cerebral magnetic resonance spectroscopy in clinical studies. However, validation of Lac/NAA ratios as a surrogate biomarker in preclinical studies is lacking.

None of the ongoing clinical trials identified are recruiting in low- and middle-income countries where therapeutic hypothermia is not an option although bulk of the burden of neonatal HIE is in this region. All treatment strategies discussed above except for possibly melatonin can be administered without intensive care support. Further preclinical studies in the context of infection-inflammation are required before such trials are undertaken.

## Conclusion

A wide variety of experimental treatment approaches for neonatal HIE have progressed towards the clinic over recent years. There is a need for more optimal animal models, greater industry support/sponsorship, increased use of juvenile toxicology, dose-ranging studies with pharmacokinetic-pharmacodynamic modelling, and well-designed clinical trials to avoid exposure to harmful medications or abandoning putative treatments.

## Abbreviations

aEEG: Amplitude-integrated electroencephalography
AUC: Area under curve
CI: Confidence interval
*C*_max_: Maximum concentrations
GLP-1: Glucagon-like peptide-1
HIE: Hypoxic-ischemic encephalopathy
Lac/NAA: Lactate/N-acetyl aspartate ratio
MRI: Magnetic resonance imaging
MRS: Magnetic resonance spectroscopy
NNT: Numbers needed to treat
NOAEL: No adverse observed adverse event level
PPAR: Peroxisome proliferator-activated receptor
RR: Risk ratio
SD: Standard deviation
TH: Therapeutic hypothermia
TUNEL: Terminal deoxynucleotidyl transferase dUTP nick end labelling

## References

[1] Kurinczuk JJ, White-Koning M, Badawi N (2010). Epidemiology of neonatal encephalopathy and hypoxic-ischaemic encephalopathy. Early Hum Dev.

[2] Gale C, Statnikov Y, Jawad S, Uthaya SN, Modi N (2018). Neonatal brain injuries in England: population-based incidence derived from routinely recorded clinical data held in the National Neonatal Research Database. Arch Dis Child Fetal Neonatal Ed.

[3] Lee ACC, Kozuki N, Blencowe H, Vos T (2013). Intrapartum-related neonatal encephalopathy incidence and impairment at regional and global levels for 2010 with trends from 1990. Pediatr Res.

[4] Edwards AD, Brocklehurst P, Gunn AJ, Halliday H, Juszczak E, Levene M, Strohm B, Thoresen M, Whitelaw A, Azzopardi D (2010). Neurological outcomes at 18 months of age after moderate hypothermia for perinatal hypoxic ischaemic encephalopathy: synthesis and meta-analysis of trial data. BMJ (Online).

[5] Tagin MA, Woolcott CG, Vincer MJ, Whyte RK, Stinson DA (2012). Hypothermia for neonatal hypoxic ischemic encephalopathy: an updated systematic review and meta-analysis. Arch Pediatr Adolesc Med.

[6] Oliveira V, Raman Kumutha J, Somanna J (2018). Hypothermia for encephalopathy in low-income and middle-income countries: feasibility of whole-body cooling using a low-cost servo-controlled device on behalf of HELIX consortium. BMJ Paediatr Open.

[7] Thayyil S, Pant S, Montaldo P (2021). Hypothermia for moderate or severe neonatal encephalopathy in low-income and middle-income countries (HELIX): a randomised controlled trial in India, Sri Lanka, and Bangladesh. Lancet Glob Health.

[8] Nagai A, Nakagawa E, Choi HB, Hatori K, Kobayashi S, Kim SU (2001). Erythropoietin and erythropoietin receptors in human CNS neurons, astrocytes, microglia, and oligodendrocytes grown in culture. J Neuropathol Exp Neurol.

[9] Yamaji R, Okada T, Moriya M, Naito M, Tsuruo T, Miyatake K, Nakano Y (1996). Brain capillary endothelial cells express two forms of erythropoietin receptor mRNA. Eur J Biochem.

[10] Yu X, Shacka JJ, Eells JB, Suarez-Quian C (2002). Erythropoietin receptor signalling is required for normal brain development. Development.

[11] Juul SE, Yachnis AT, Rojiani AM, Christensen RD (1999). Immunohistochemical localization of erythropoietin and its receptor in the developing human brain. Pediatr Dev Pathol.

[12] Gonzalez FF, Larpthaveesarp A, McQuillen P, Derugin N, Wendland M, Spadafora R, Ferriero DM (2013). Erythropoietin increases neurogenesis and oligodendrogliosis of subventricular zone precursor cells after neonatal stroke. Stroke.

[13] Kaneko N, Kako E, Sawamoto K (2013). Enhancement of ventricular-subventricular zone-derived neurogenesis and oligodendrogenesis by erythropoietin and its derivatives. Front Cell Neurosci.

[14] Xiong Y, Mahmood A, Meng Y, Zhang Y, Qu C, Schallert T, Chopp M (2010). Delayed administration of erythropoietin reducing hippocampal cell loss, enhancing angiogenesis and neurogenesis, and improves functional outcome following traumatic brain injury in rats: comparison of treatment with single and triple dose. J Neurosurg.

[15] Ikeda E (2005). Cellular response to tissue hypoxia and its involvement in disease progression. Pathol Int.

[16] Villa P, van Beek J, Larsen AK, Gerwien J, Christensen S, Cerami A, Brines M, Leist M, Ghezzi P, Torup L (2007). Reduced functional deficits, neuroinflammation, and secondary tissue damage after treatment of stroke by nonerythropoietic erythropoietin derivatives. J Cereb Blood Flow Metab.

[17] Juul SE, Beyer RP, Bammler TK, Mcpherson RJ, Wilkerson J, Farin FM (2009). Microarray analysis of high-dose recombinant erythropoietin treatment of unilateral brain injury in neonatal mouse hippocampus. Pediatr Res.

[18] Wang L, Zhang ZG, Zhang RL, Gregg SR, Hozeska-Solgot A, LeTourneau Y, Wang Y, Chopp M (2006). Matrix metalloproteinase 2 (MMP2) and MMP9 secreted by erythropoietin-activated endothelial cells promote neural progenitor cell migration. J Neurosci.

[19] Iwai M, Cao G, Yin W, Stetler RA, Liu J, Chen J (2007). Erythropoietin promotes neuronal replacement through revascularization and neurogenesis after neonatal hypoxia/ischemia in rats. Stroke.

[20] Juul SE, McPherson RJ, Bammler TK, Wilkerson J, Beyer RP, Farin FM (2008). Recombinant erythropoietin is neuroprotective in a novel mouse oxidative injury model. Dev Neurosci.

[21] Fan X, Heijnen CJ, van der Kooij MA, Groenendaal F, van Bel F (2011). Beneficial effect of erythropoietin on sensorimotor function and white matter after hypoxia-ischemia in neonatal mice. Pediatr Res.

[22] Larpthaveesarp A, Georgevits M, Ferriero DM, Gonzalez FF (2016). Delayed erythropoietin therapy improves histological and behavioral outcomes after transient neonatal stroke. Neurobiol Dis.

[23] van de Looij Y, Chatagner A, Quairiaux C, Gruetter R, Hüppi PS, Sizonenko S (2014) Multi-modal assessment of long-term erythropoietin treatment after neonatal hypoxic-ischemic injury in rat brain. PLoS One 9:e95643. 10.1371/journal.pone.0095643 10.1371/journal.pone.0095643 PMC3995802 24755676

[24] Traudt CM, Mcpherson RJ, Bauer LA, Richards TL, Burbacher TM, Mcadams RM, Juul SE (2013). Concurrent erythropoietin and hypothermia treatment improve outcomes in a term nonhuman primate model of perinatal asphyxia. Dev Neurosci.

[25] McAdams RM, Fleiss B, Traudt C, Schwendimann L, Snyder JM, Haynes RL, Natarajan N, Gressens P, Juul SE (2017). Long-term neuropathological changes associated with cerebral palsy in a nonhuman primate model of hypoxic-ischemic encephalopathy. Dev Neurosci.

[26] Pang R, Avdic-Belltheus A, Meehan C et al (2021) Melatonin and/or erythropoietin combined with hypothermia in a piglet model of perinatal asphyxia. Brain Commun 3:fcaa211. 10.1093/braincomms/fcaa211 10.1093/braincomms/fcaa211 PMC7876304 33604569

[27] Oorschot DE, Sizemore RJ, Amer AR (2020). Treatment of neonatal hypoxic-ischemic encephalopathy with erythropoietin alone, and erythropoietin combined with hypothermia: history, current status, and future research. Int J Mol Sci.

[28] Wu YW, Bauer LA, Ballard RA, Ferriero DM (2012). Erythropoietin for neuroprotection in neonatal encephalopathy: safety and pharmacokinetics. Pediatrics.

[29] Frymoyer A, Juul SE, Massaro AN, Bammler TK, Wu YW (2017). High-dose erythropoietin population pharmacokinetics in neonates with hypoxic-ischemic encephalopathy receiving hypothermia. Pediatr Res.

[30] Malla RR, Asimi R, Teli MA, Shaheen F, Bhat MA (2017). Erythropoietin monotherapy in perinatal asphyxia with moderate to severe encephalopathy: a randomized placebo-controlled trial. J Perinatol.

[31] Baserga MC, Beachy JC, Roberts JK (2015). Darbepoetin administration to neonates undergoing cooling for encephalopathy: a safety and pharmacokinetic trial. Pediatr Res.

[32] Rogers EE, Bonifacio SL, Glass HC (2014). Erythropoietin and hypothermia for hypoxic-ischemic encephalopathy. Pediatr Neurol.

[33] Nonomura M, Harada S, Asada Y, Matsumura H, Iwami H, Tanaka Y, Ichiba H (2019). Combination therapy with erythropoietin, magnesium sulfate and hypothermia for hypoxic-ischemic encephalopathy: an open-label pilot study to assess the safety and feasibility. BMC Pediatr.

[34] Mulkey SB, Ramakrishnaiah RH, McKinstry RC (2017). Erythropoietin and brain magnetic resonance imaging findings in hypoxic-ischemic encephalopathy: volume of acute brain injury and 1-year neurodevelopmental outcome. J Pediatr.

[35] El Shimi MS, Awad HA, Hassanein SMA, Gad GI, Imam SS, Shaaban HA, El Maraghy MO (2014). Single dose recombinant erythropoietin versus moderate hypothermia for neonatal hypoxic ischemic encephalopathy in low resource settings. J Matern Fetal Neonatal Med.

[36] Avasiloaiei A, Dimitriu C, Moscalu M, Paduraru L, Stamatin M (2013). High-dose phenobarbital or erythropoietin for the treatment of perinatal asphyxia in term newborns. Pediatr Int.

[37] Elmahdy H, El-Mashad AR, El-Bahrawy H, El-Gohary T, El-Barbary A, Aly H (2010). Human recombinant erythropoietin in asphyxia neonatorum: pilot trial. Pediatrics.

[38] Zhu C, Kang W, Xu F (2009). Erythropoietin improved neurologic outcomes in newborns with hypoxic-ischemic encephalopathy. Pediatrics.

[39] Wu YW, Mathur AM, Chang T (2016). High-dose erythropoietin and hypothermia for hypoxic-ischemic encephalopathy: a phase II trial. Pediatrics.

[40] Juul SE, Comstock BA, Heagerty PJ, Mayock DE, Goodman AM, Hauge S, Gonzalez F, Wu YW (2018). High-dose erythropoietin for asphyxia and encephalopathy (HEAL): a randomized controlled trial-background, aims, and study protocol. Neonatology.

[41] Tordjman S, Chokron S, Delorme R, Charrier A, Bellissant E, Jaafari N, Fougerou C (2017). Melatonin: pharmacology, functions and therapeutic benefits. Curr Neuropharmacol.

[42] Sagrillo-Fagundes L, Maria Assuncao Salustiano E, Wong Yen P, Soliman A, Vaillancourt C (2016). Melatonin in pregnancy: effects on brain development and CNS programming disorders. Curr Pharm Des.

[43] Drew JE, Williams LM, Hannah LT, Barrett P, Abramovich DR, Morgan PJ (1997). Identification and characterisation of 2-[125I]iodomelatonin binding and Mel1a melatonin receptor expression in the human fetal leptomeninges. Brain Res.

[44] Thomas L, Purvis C, Drew JE, Abramovich R, Williams LM (2002). Melatonin receptors in human fetal brain: 2-[125I]iodomelatonin binding and MT1 gene expression. J Pineal Res.

[45] Okatani Y, Wakatsuki A, Kaneda C (2000). Melatonin increases activities of glutathione peroxidase and superoxide dismutase in fetal rat brain. J Pineal Res.

[46] Chitimus DM, Popescu MR, Voiculescu SE, Panaitescu AM, Pavel B, Zagrean L, Zagrean A-M (2020). Melatonin’s impact on antioxidative and anti-inflammatory reprogramming in homeostasis and disease. Biomolecules.

[47] Wakatsuki A, Okatani Y, Shinohara K, Ikenoue N, Fukaya T (2001). Melatonin protects against ischemia/reperfusion-induced oxidative damage to mitochondria in fetal rat brain. J Pineal Res.

[48] Robertson NJ, Faulkner S, Fleiss B (2013). Melatonin augments hypothermic neuroprotection in a perinatal asphyxia model. Brain.

[49] Robertson NJ, Lingam I, Meehan C (2020). High-dose melatonin and ethanol excipient combined with therapeutic hypothermia in a newborn piglet asphyxia model. Sci Rep.

[50] Robertson NJ, Martinello K, Lingam I (2019). Melatonin as an adjunct to therapeutic hypothermia in a piglet model of neonatal encephalopathy: a translational study. Neurobiol Dis.

[51] Aridas JDS, Yawno T, Sutherland AE (2018). Systemic and transdermal melatonin administration prevents neuropathology in response to perinatal asphyxia in newborn lambs. J Pineal Res.

[52] Pang R, Advic-Belltheus A, Meehan C, Fullen DJ, Golay X, Robertson NJ (2021). Melatonin for neonatal encephalopathy: from bench to bedside. Int J Mol Sci.

[53] Gringras P, Nir T, Breddy J, Frydman-Marom A, Findling RL (2017). Efficacy and safety of pediatric prolonged-release melatonin for insomnia in children with autism spectrum disorder. J Am Aca Child Adolesc Psychiatry.

[54] Alers NO, Jenkin G, Miller SL, Wallace EM (2013). Antenatal melatonin as an antioxidant in human pregnancies complicated by fetal growth restriction - a phase I pilot clinical trial: study protocol. BMJ Open.

[55] Hobson SR, Lim R, Gardiner EE, Alers NO, Wallace EM (2013). Phase I pilot clinical trial of antenatal maternally administered melatonin to decrease the level of oxidative stress in human pregnancies affected by pre-eclampsia (PAMPR): study protocol. BMJ Open.

[56] Hobson SR, Gurusinghe S, Lim R, Alers NO, Miller SL, Kingdom JC, Wallace EM (2018). Melatonin improves endothelial function in vitro and prolongs pregnancy in women with early-onset preeclampsia. J Pineal Res.

[57] Carloni S, Proietti F, Rocchi M, Longini M, Marseglia L, D’Angelo G, Balduini W, Gitto E, Buonocore G (2017). Melatonin pharmacokinetics following oral administration in preterm neonates. Molecules.

[58] Aly H, Elmahdy H, El-Dib M, Rowisha M, Awny M, El-Gohary T, Elbatch M, Hamisa M, El-Mashad AR (2015). Melatonin use for neuroprotection in perinatal asphyxia: a randomized controlled pilot study. J Perinatol.

[59] El Farargy MS, Soliman NA (2020). A randomized controlled trial on the use of magnesium sulfate and melatonin in neonatal hypoxic ischemic encephalopathy. J Neonatal Perinatal Med.

[60] Ahmad QM, Chishti AL, Waseem N (2018). Role of melatonin in management of hypoxic ischaemic encephalopathy in newborns: a randomized control trial. J Pak Med Assoc.

[61] Fulia F, Gitto E, Cuzzocrea S, Reiter RJ, Dugo L, Gitto P, Barberi S, Cordaro S, Barberi I (2001). Increased levels of malondialdehyde and nitrite/nitrate in the blood of asphyxiated newborns: reduction by melatonin. J Pineal Res.

[62] Jerez-Calero A, Salvatierra-Cuenca MT, Benitez-Feliponi Á, Fernández-Marín CE, Narbona-López E, Uberos-Fernández J, Munõz-Hoyos A (2020). Hypothermia plus melatonin in asphyctic newborns: a randomized-controlled pilot study. Pediatr Crit Care Med.

[63] Zou S, Kumar U (2018). Cannabinoid receptors and the endocannabinoid system: signaling and function in the central nervous system. Int J Mol Sci.

[64] Martínez-Orgado J, Villa M, del Pozo A (2021). Cannabidiol for the treatment of neonatal hypoxic-ischemic brain injury. Front Pharmacol.

[65] Campos AC, Fogaça MV, Sonego AB, Guimarães FS (2016). Cannabidiol, neuroprotection and neuropsychiatric disorders. Pharmacol Res.

[66] Pazos MR, Mohammed N, Lafuente H (2013). Mechanisms of cannabidiol neuroprotection in hypoxic-ischemic newborn pigs: role of 5HT1A and CB2 receptors. Neuropharmacology.

[67] Castillo A, Tolón MR, Fernández-Ruiz J, Romero J, Martinez-Orgado J (2010). The neuroprotective effect of cannabidiol in an in vitro model of newborn hypoxic-ischemic brain damage in mice is mediated by CB2 and adenosine receptors. Neurobiol Dis.

[68] Franco R, Villa M, Morales P, Reyes-Resina I, Gutiérrez-Rodríguez A, Jiménez J, Jagerovic N, Martínez-Orgado J, Navarro G (2019). Increased expression of cannabinoid CB2 and serotonin 5-HT1A heteroreceptor complexes in a model of newborn hypoxic-ischemic brain damage. Neuropharmacology.

[69] Alonso-Alconada D, Álvarez A, Álvarez FJ, Martínez-Orgado JA, Hilario E (2012). The cannabinoid WIN 55212–2 mitigates apoptosis and mitochondrial dysfunction after hypoxia ischemia. Neurochem Res.

[70] Alonso-Alconada D, Álvarez FJ, Goñi-De-cerio F, Hilario E, Álvarez A (2020). Cannabinoid-mediated modulation of oxidative stress and early inflammatory response after hypoxia–ischemia. Int J Mol Sci.

[71] Patricio F, Morales-Andrade AA, Patricio-Martínez A, Limón ID (2020). Cannabidiol as a therapeutic target: evidence of its neuroprotective and neuromodulatory function in Parkinson’s disease. Front Pharmacol.

[72] Barata L, Arruza L, Rodríguez MJ (2019). Neuroprotection by cannabidiol and hypothermia in a piglet model of newborn hypoxic-ischemic brain damage. Neuropharmacology.

[73] Lafuente H, Pazos MR, Alvarez A, Mohammed N, Santos M, Arizti M, Alvarez FJ, Martinez-Orgado JA (2016). Effects of cannabidiol and hypothermia on short-term brain damage in new-born piglets after acute hypoxia-ischemia. Front Neurosci.

[74] Garberg HT, Huun MU, Escobar J, Martinez-Orgado J, Løberg E-M, Solberg R, Saugstad OD (2016). Short-term effects of cannabidiol after global hypoxia-ischemia in newborn piglets. Pediatr Res.

[75] Alonso-Alconada D, Alvarez FJ, Alvarez A, Mielgo VE, Goñi-De-Cerio F, Rey-Santano MC, Caballero A, Martinez-Orgado J, Hilario E (2010). The cannabinoid receptor agonist WIN 55,212–2 reduces the initial cerebral damage after hypoxic-ischemic injury in fetal lambs. Brain Res.

[76] Mohammed N, Ceprian M, Jimenez L, Pazos M, Martínez-Orgado J (2016). Neuroprotective effects of cannabidiol in hypoxic ischemic insult. The therapeutic window in newborn mice. CNS Neurol Disord Drug Targets.

[77] Mechoulam R, Parker LA, Gallily R (2002). Cannabidiol: an overview of some pharmacological aspects. J Clin Pharmacol.

[78] Millar SA, Stone NL, Yates AS, O’Sullivan SE (2018). A systematic review on the pharmacokinetics of cannabidiol in humans. Front Pharmacol.

[79] Ohlsson A, Lindgren J-E, Andersson S, Agurell S, Gillespie H, Hollister LE (1986). Single-dose kinetics of deuterium-labelled cannabidiol in man after smoking and intravenous administration. Biomed Environ Mass Spectrom.

[80] Thiele EA, Marsh ED, French JA (2018). Cannabidiol in patients with seizures associated with Lennox-Gastaut syndrome (GWPCARE4): a randomised, double-blind, placebo-controlled phase 3 trial. Lancet.

[81] Devinsky O, Nabbout R, Miller I, Laux L, Zolnowska M, Wright S, Roberts C (2019). Long-term cannabidiol treatment in patients with Dravet syndrome: an open-label extension trial. Epilepsia.

[82] Paternoster S, Falasca M (2018). Dissecting the physiology and pathophysiology of glucagon-like peptide-1. Front Endocrinol.

[83] Jia XT, Ye-Tian Y-L (2016). Exendin-4, a glucagon-like peptide 1 receptor agonist, protects against amyloid-β peptide-induced impairment of spatial learning and memory in rats. Physiol Behav.

[84] Foltynie T, Aviles-Olmos I (2014). Exenatide as a potential treatment for patients with Parkinson’s disease: first steps into the clinic. Alzheimers Dement.

[85] Eakin K, Li Y, Chiang Y-H, Hoffer BJ, Rosenheim H, Greig NH, Miller JP (2013) Exendin-4 ameliorates traumatic brain injury-induced cognitive impairment in rats. PLoS One 8:82016. 10.1371/journal.pone.0082016 10.1371/journal.pone.0082016 PMC3847068 24312624

[86] Rachmany L, Tweedie D, Rubovitch V (2017). Exendin-4 attenuates blast traumatic brain injury induced cognitive impairments, losses of synaptophysin and in vitro TBI-induced hippocampal cellular degeneration. Sci Rep.

[87] Marlet IR, Ölmestig JNE, Vilsbøll T, Rungby J, Kruuse C (2018). Neuroprotective mechanisms of glucagon-like peptide-1-based therapies in ischaemic stroke: a systematic review based on pre-clinical studies. Basic Clin Pharmacol Toxicol.

[88] Teramoto S, Miyamoto N, Yatomi K, Tanaka Y, Oishi H, Arai H, Hattori N, Urabe T (2011). Exendin-4, a glucagon-like peptide-1 receptor agonist, provides neuroprotection in mice transient focal cerebral ischemia. J Cereb Blood Flow Metab.

[89] Zeng S, Bai J, Jiang H (2020). Treatment with liraglutide exerts neuroprotection after hypoxic-ischemic brain injury in neonatal rats via the PI3K/AKT/GSK3β pathway. Front Cell Neurosci.

[90] Wang MD, Huang Y, Zhang GP, Mao L, Xia YP, Mei YW, Hu B (2012). Exendin-4 improved rat cortical neuron survival under oxygen/glucose deprivation through PKA pathway. Neuroscience.

[91] Zhu H, Zhang Y, Shi Z, Lu D, Li T, Ding Y, Ruan Y, Xu A (2016). The neuroprotection of liraglutide against ischaemia-induced apoptosis through the activation of the PI3K/AKT and MAPK pathways. Sci Rep.

[92] Rocha-Ferreira E, Poupon L, Zelco A, Leverin AL, Nair S, Jonsdotter A, Carlsson Y, Thornton C, Hagberg H, Rahim AA (2018). Neuroprotective exendin-4 enhances hypothermia therapy in a model of hypoxic-ischaemic encephalopathy. Brain.

[93] Cirincione B, Mager DE (2017). Population pharmacokinetics of exenatide. Br J Clin Pharmacol.

[94] Copley K, McCowen K, Hiles R, Nielsen L, Young A, Parkes D (2006). Investigation of exenatide elimination and its in vivo and in vitro degradation. Curr Drug Metab.

[95] van Meijel LA, Rooijackers HM, Tack CJ, de Galan BE (2019). Effect of the GLP-1 receptor agonist exenatide on impaired awareness of hypoglycemia in type 1 diabetes: a randomized controlled trial. J Clin Endocrinol Metab.

[96] Calara F, Taylor K, Han J, Zabala E, Moo Carr E, Wintle M, Fineman M (2005). A randomized, open-label, crossover study examining the effect of injection site on bioavailability of exenatide (synthetic exendin-4). Clin Ther.

[97] Krishnan L, Dhatariya K, Gerontitis D (2013). No clinical harm from a massive exenatide overdose-a short report. Clin Toxicol.

[98] Cohen V, Teperikidis E, Jellinek SP, Rose J (2009). Acute exenatide (Byetta®) poisoning was not associated with significant hypoglycemia. Clin Toxicol.

[99] Annink KV, Franz AR, Derks JB, Rüdiger M, van Bel F, Benders MJNL (2018). Allopurinol: old drug, new indication in neonates?. Curr Pharm Des.

[100] Palmer C, Towfighi J, Roberts RL, Heitjan DF (1993). Allopurinol administered after inducing hypoxia-ischemia reduces brain injury in 7-day-old rats. Pediatr Res.

[101] Williams GD, Palmer C, Heitjan DF, Smith MB (1992). Allopurinol preserves cerebral energy metabolism during perinatal hypoxia-ischemia: a 31P NMR study in unanesthetized immature rats. Neurosci Lett.

[102] Palmer C, Vannucci RC, Towfighi J (1990). Reduction of perinatal hypoxic-ischemic brain damage with allopurinol. Pediatr Res.

[103] Peeters-Scholte C, van den Tweel E, Ioroi T, Post I, Braun K, Veldhuis W, Nicolay K, Groenendaal F, van Bel F (2002). Pharmacological interventions in the newborn piglet in the first 24 h after hypoxia-ischemia: a hemodynamic and electrophysiological perspective. Exp Brain Res.

[104] Peeters-Scholte C, Braun K, Koster J (2003). Effects of allopurinol and deferoxamine on reperfusion injury of the brain in newborn piglets after neonatal hypoxia-ischemia. Pediatr Res.

[105] Marro PJ, Hoffman D, Schneiderman R, Mishra OP, Delivoria-Papadopoulos M (1998). Effect of allopurinol on NMDA receptor modification following recurrent asphyxia in newborn piglets. Brain Res.

[106] Marro PJ, Mishra OP, Delivoria-Papadopoulos M (2006). Effect of allopurinol on brain adenosine levels during hypoxia in newborn piglets. Brain Res.

[107] Maiwald CA, Annink KV, Rüdiger M (2019). Effect of allopurinol in addition to hypothermia treatment in neonates for hypoxic-ischemic brain injury on neurocognitive outcome (ALBINO): study protocol of a blinded randomized placebo-controlled parallel group multicenter trial for superiority (phase III). BMC Pediatr.

[108] Gunes T, Ozturk MA, Koklu E, Kose K, Gunes I (2007). Effect of allopurinol supplementation on nitric oxide levels in asphyxiated newborns. Pediatr Neurol.

[109] Chu WY, Annink KV, Nijstad AL (2021). Pharmacokinetic/pharmacodynamic modelling of allopurinol, its active metabolite oxypurinol, and biomarkers hypoxanthine, xanthine and uric acid in hypoxic-ischemic encephalopathy neonates. Clin Pharmacokinet.

[110] Chaudhari T, McGuire W (2012) Allopurinol for preventing mortality and morbidity in newborn infants with hypoxic-ischaemic encephalopathy. Cochrane Database Syst Rev 11:CD006817. 10.1002/14651858.cd006817.pub3 10.1002/14651858.CD006817.pub3 PMC11260067 22786499

[111] Serrenho I, Rosado M, Dinis A, Cardoso CM, Grãos M, Manadas B, Baltazar G (2021). Stem cell therapy for neonatal hypoxic-ischemic encephalopathy: a systematic review of preclinical studies. Int J Mol Sci.

[112] Bruschettini M, Romantsik O, Moreira A, Ley D, Thébaud B (2020) Stem cell-based interventions for the prevention of morbidity and mortality following hypoxic-ischaemic encephalopathy in newborn infants. Cochrane Database Syst Rev 8:CD013202. 10.1002/14651858.CD013202.pub2 10.1002/14651858.CD013202.pub2 PMC7438027 32813884

[113] Azzopardi D, Robertson NJ, Bainbridge A (2016). Moderate hypothermia within 6 h of birth plus inhaled xenon versus moderate hypothermia alone after birth asphyxia (TOBY-Xe): a proof-of-concept, open-label, randomised controlled trial. Lancet Neurol.

[114] Lingam I, Meehan C, Avdic-Belltheus A, Martinello K, Hristova M, Kaynezhad P, Bauer C, Tachtsidis I, Golay X, Robertson NJ (2019). Short-term effects of early initiation of magnesium infusion combined with cooling after hypoxia–ischemia in term piglets. Pediatr Res.

[115] Kyng KJ, Skajaa T, Kerrn-Jespersen S, Andreassen CS, Bennedsgaard K, Henriksen TB (2015). A piglet model of neonatal hypoxic-ischemic encephalopathy. J Vis Exp.

[116] European Medicines Agency: EMA/CHMP/272866/2013 (2017) Benzyl alcohol and benzoic acid group used as excipients. In: https://www.ema.europa.eu/en/documents/report/benzyl-alcohol-benzoic-acid-group-usedexcipients-report-published-support-questions-answers-benzyl/chmp/508188/2013-t_en.pdf.www.ema.europa.eu/contact

[117] Weber C, Kammerer D, Streit B, Licht AH (2015) Phenolic excipients of insulin formulations induce cell death, pro-inflammatory signaling and MCP-1 release. Toxicol Rep 2:194–202. 10.1016/j.toxrep.2014.11.019 10.1016/j.toxrep.2014.11.019 PMC5598374 28962351

[118] U.S. Department of Health and Human Services (2005) Guidance for Industry: estimating the maximum safe starting dose in initial clinical trials for therapeutics in adult healthy volunteers. In: https://www.fda.gov/media/72309/download

